# Randomized, double-blind, placebo-controlled, parallel-group trial of sirolimus for tocilizumab-resistant idiopathic multicentric Castleman disease: Study protocol for clinical trial

**DOI:** 10.1097/MD.0000000000020710

**Published:** 2020-07-24

**Authors:** Tomohiro Koga, Naoko Hagimori, Sachiko Takemori, Shimpei Morimoto, Remi Sumiyoshi, Toshimasa Shimizu, Naoki Hosogaya, Chizu Fukushima, Hiroshi Yamamoto, Atsushi Kawakami

**Affiliations:** a Department of Immunology and Rheumatology, Division of Advanced Preventive Medical Sciences; b Center for Bioinformatics and Molecular Medicine, Nagasaki University Graduate School of Biomedical Sciences; c Nagasaki University Hospital, Clinical Research Center, Nagasaki, Japan.

**Keywords:** double-blind, idiopathic multicentric Castleman disease, mammalian target of rapamycin, sirolimus, tocilizumab-resistant

## Abstract

**Background:**

Idiopathic multicentric Castleman disease (iMCD) is a rare lymphoproliferative disorder of unknown etiology with systemic symptoms that include fever, night sweats, weight loss, and fatigue. Although tocilizumab (TCZ), which is a recombinant, humanized, anti-human interleukin 6 receptor monoclonal antibody, has been recommended to treat patients with iMCD, 40% of patients with iMCD do not achieve complete remission with TCZ treatment.

**Methods/Design:**

In this phase II, investigator-initiated, multicenter, double-blind, randomized, parallel-group trial, the efficacy and safety of sirolimus will be compared with placebo in patients with TCZ-resistant iMCD. The study will be conducted in 8 centers in Japan. Participants (n = 20) will be randomly assigned to receive 2 mg of oral sirolimus (n = 10) or placebo (n = 10) once daily for 16 weeks. The primary endpoint is a decrease in CHAP score by ≥1 from baseline at 16 weeks. Secondary endpoints include levels of hemoglobin, albumin, and C-reactive protein; change in CHAP score; SF-36 Health Survey Questionnaire; physician global assessment (100 mm visual analog scale); patient global assessment (100 mm visual analog scale) at 2, 4, 8, 12, and 16 weeks; change in lymphadenopathy at 16 weeks; and pharmacodynamic assessment, including the measurement of whole blood sirolimus level.

**Discussion:**

This clinical trial will provide evidence of efficacy and safety of sirolimus as a potential new therapeutic agent for patients with TCZ-resistant iMCD.

**Trial Registration:**

This study was registered with the Japan Registry of Clinical Trials as jRCT2071190029 on October 8, 2019.

## 1 Introduction

Castleman disease (CD) is a rare lymphoproliferative disorder with 2 distinct clinical entities: the localized form, unicentric Castleman disease and the multicentric form, multicentric CD (MCD).^[1,2]^ Individuals with unicentric Castleman disease are generally asymptomatic, whereas MCD is a systemic disease with multiple regions of lymphadenopathy and systemic symptoms that include fever, night sweats, weight loss, and fatigue.^[3–5]^ Some MCD cases are associated with human herpesvirus 8 infection in human immunodeficiency virus-positive patients.^[6]^ However, for patients with unknown etiology and pathophysiology, MCD is referred to as idiopathic MCD (iMCD).^[7,8]^

In clinical practice, therapy for iMCD is introduced to improve systemic symptoms, reduce the size of lymph nodes, normalize levels of acute-phase reactants such as C-reactive protein (CRP), and to prevent organ damage. The anti-interleukin 6 (IL-6) monoclonal antibody including tocilizumab (TCZ) is the preferred first-line therapy for iMCD.^[9]^ However, 40% of iMCD cases are refractory or resistant to TCZ.^[10]^ In addition, plasma proteomics and histological examination of lymph nodes suggest that an IL-6 independent pathway exists for the pathogenesis of iMCD.^[11]^

Sirolimus, a mammalian target of rapamycin (mTOR) inhibitor, has been reported to be effective for iMCD cases that are refractory to IL-6 inhibitors,^[12]^ suggesting activation of the phosphatidylinositol-3 kinase/Akt/mTOR pathway in the pathogenesis of iMCD. Inhibition of this pathway suppresses proliferation of T cells and B cells activated by iMCD, as well as vascular endothelial growth factor, and is especially effective for some IL -6 independent iMCD.^[13,14]^ In a recent report of 3 cases, sirolimus treatment significantly attenuated CD8-positive T cell activation and decreased vascular endothelial growth factor-A levels in patients with IL-6 inhibitor-resistant iMCD and achieved clinical remission in all 3 cases.^[15]^ Thus, sirolimus may be a candidate for second-line therapy in patients with inadequate response to TCZ.

Taken together, these findings prompted us to design the current phase II study to confirm the beneficial effects of sirolimus in patients with iMCD. Herein, we describe the final protocol (version 3.1; April 19, 2019) for this study. The results of this study are expected to provide evidence regarding the usefulness of sirolimus for the treatment of TCZ-resistant iMCD patients.

## 2 Methods/design

### 2.1 Study design

The present study has been designed in accordance with the standard protocol items: Recommendations for Interventional Trials and Consolidated Standards of Reporting Trials 2010 guidelines.^[16,17]^ This is an investigator-initiated, multicenter, phase II, double-blind, randomized, parallel-group comparison study of the efficacy and safety of sirolimus compared with placebo in patients with TCZ-resistant iMCD.

This study will be conducted at 8 centers in Japan and will be performed in accordance with the principles of the Declaration of Helsinki^[18]^ and the Japan Good Clinical Practice. The study was registered on the Japan Registry of Clinical Trials as jRCT2071190029, and was approved by the Institutional Review Board in Nagasaki University Hospital, Keio University Hospital, Jikei University Hospital, Kanazawa Medical University Hospital, Kyoto University Hospital, Sumitomo Hospital, Daini Osaka Police Hospital, and Kyushu University Hospital.

### 2.2 Participant recruitment

Participants will be recruited at Nagasaki University Hospital, Keio University Hospital, Jikei University Hospital, Kanazawa Medical University Hospital, Kyoto University Hospital, Sumitomo Hospital, Daini Osaka Police Hospital, and Kyushu University Hospital. All eligible patients will be selected and approached based on information from the electronic health record from these 8 hospitals according to the inclusion and exclusion criteria. In addition, we emailed the chief of the department in charge of potential patients describing the outline of this clinical trial using the mailing list of the Castleman Disease Regional Hospital Network in Japan. The guidance on the recruitment was advertised in the home page of intractable disease research support foundation and intractable disease information center. Participants will be provided with an explanation regarding the study by their treating pediatrician/rheumatologist and clinical research coordinator and will be asked to voluntarily sign an informed consent form before their participation.

### 2.3 Inclusion criteria

Patients who meet the following inclusion criteria are eligible for this study.

1) Patients diagnosed as having iMCD according to the Japanese diagnostic criteria (“regarding diagnostic criteria and severity classification related to designated intractable diseases” by the Ministry of Health, Labor and Welfare in Japan).^[10]^
2) Patients aged 18 years or older at the time of obtaining consent (regardless of sex).
3) Patients with 1 or more points of CRP, hemoglobin (Hb), or albumin (Alb) as defined by the CHAP score^[10]^ despite TCZ treatment for more than 8 weeks.
4) Patients who continued corticosteroids (prednisolone equivalent 0.5 mg/kg/d or less) at the same dose for at least 4 weeks from the time consent was obtained.
5) Patients who received a thorough explanation of the contents of explanatory documents and other matters concerning clinical trials, understood the contents thereof, and provided written consent based on their free will to participate in this trial.

### 2.4 Exclusion criteria

Patients with any of the following at the time of screening will be excluded:

1) Neutrophil count <1000/μL.
2) Platelet count <75,000/μL.
3) Alanine transaminase exceeding 3.0 times the upper limit of normal.
4) Aspartate aminotransferase exceeding 3.0 times the upper limit of normal.
5) Total bilirubin >1.5 times the baseline.
6) Alkaline phosphatase exceeding 2.5 times the upper limit of the reference value.
7) Serum creatinine >1.5 times the upper limit of normal and creatinine clearance <50 mL/min. Estimated creatinine clearance is determined using the Cockcroft-Gault formula. However, if there is an actual measurement, the actual measurement is used.
8) Patients with uncontrolled dyslipidemia. Serum triglyceride level of >500 mg/dL or an LDL cholesterol level of ≥190 mg/dL who are receiving treatment for dyslipidemia.
9) Patients with poorly controlled diabetes mellitus. Diabetic patients with fasting blood glucose <130 mg/dL or HbA1c ≤8.0%, despite being treated for diabetes.
10) Patients with a history of myocardial infarction, angina pectoris, or stroke associated with atherosclerosis.
11) Patients with an Eastern Cooperative Oncology Group Performance Status of 4.
12) Patients with concomitant or a history of idiopathic pulmonary fibrosis or drug-induced pulmonary disorder.
13) Patients with active tuberculosis.
14) Patients who have complicated infectious diseases within 4 weeks of the first administration of the investigational drug and who are judged to be inappropriate by the investigator or subinvestigator.
15) Patients who were diagnosed with a malignancy within 5 years before the initial administration of the study drug. This excludes cases in which resection or no treatment was performed for more than 5 years before the first administration of the investigational drug, cured skin cancer (epithelial cell carcinoma or basal cell carcinoma), and cured cervical cancer.
16) Patients with active hepatitis B, hepatitis C, or human immunodeficiency virus infection.
17) Patients complicated by a serious illness who are judged by the investigator or subinvestigator to be inappropriate for the study.
18) Patients who have previously used an mTOR inhibitor that includes sirolimus, everolimus, and temsirolimus, except when contained in drug-eluting stents or external preparations.
19) Patients with a history of hypersensitivity to any of the ingredients of the investigational drug.
20) Patients who cannot use appropriate contraception during the study drug administration period or 12 weeks after the last treatment with the study drug.
21) Female patients during lactation or pregnancy.
22) Patients who underwent surgery involving invasion of a body cavity or requiring a suture of 3 or more needles within 8 weeks before the first dose of study drug.
23) Patients who received live vaccines within 6 weeks before the initial administration of the study drug.
24) Patients who received chemotherapy within 8 weeks before the first dose of the study drug.
25) Patients who have used other investigational products or devices within 6 months before the initial administration of the investigational product.
26) Other patients deemed inappropriate by the investigator or subinvestigator.

### 2.5 Randomization

Participants will be allocated to the study group (sirolimus vs placebo) by biased coin design with imbalance tolerance.^[19]^ In the biased coin design with imbalance tolerance algorithm, the imbalance between study groups will be calculated within the following strata of the CHAP score evaluated at the randomization: score = 1, score >1. The imbalance tolerance and the biased coin probability was set based on a result from a simulation and aims to obtain treatment balance and allocation randomness. We incorporate this approach into our electronic data capture (EDC) system.

### 2.6 Study protocol

The investigator should consider whether the patient is eligible for the trial, taking into account such factors as the patient's health status, severity, and symptoms of the underlying disease and complications, age, ability to consent, and participation in other trials. They should provide sufficient information and obtain written consent from patients who are considered appropriate candidates for this trial.

The investigator should instruct the consenting patient to visit a dentist to examine the oral environment. Dental treatment should be performed within 28 days before enrollment (randomization) when the participants have dental findings associated with risk of mucositis oral. After assigning subject identification codes in the order of obtaining consent at each medical institution, the investigator will carry out the observations and examinations specified at the time of screening. Patients who meet the inclusion criteria and do not meet any of the exclusion criteria, will be randomly assigned at a rate of 1:1 to receive daily 2 mg of sirolimus or placebo subcutaneously.

**Figure 1:**
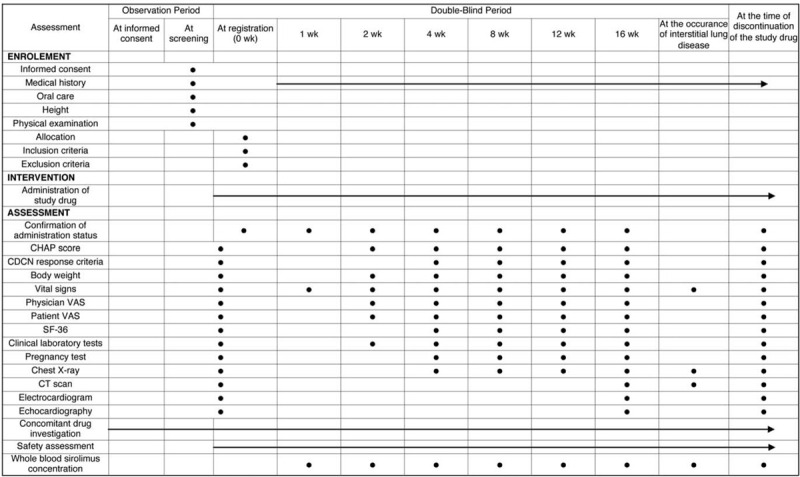
Treatment schedule and outcome measures. CDCN = Castleman Disease Collaborative Network, CT = computed tomography, ECG = electrocardiogram, VAS = visual analog scale.

After the visit date (i.e., the initial day of the investigational drug), investigators will continue to administer the investigational drug and conduct necessary examinations and surveys in accordance with the schedule shown in Figure 1. In order to prevent stomatitis, oral self-care should be performed in accordance with the stomatitis prevention and self-care guidebook created by the principal investigator. In addition, oral hygiene care and dental brushing guidance should be provided from the time of obtaining consent until completion or discontinuation of the clinical trial. If stomatitis occurs, appropriate measures should be taken, such as oral administration of topical analgesics, topical steroids for oral mucosa, and oral administration of analgesics. The dose of oral corticosteroid and TCZ should not be changed from the time of obtaining consent. We plan an open-label continuation trial of sirolimus for patients who have completed all phases of the study.

### 2.7 Adverse events

If an adverse event is observed in a patient during the study period, the investigator of each medical institution should immediately take appropriate measures and record that event in the clinical records and case reports. The severity of adverse events should be determined according to the criteria in Common Terminology Criteria for Adverse Events, version 5.0. A serious adverse event is defined as any untoward medical event that occurs at any dose, results in death, is life-threatening, requires inpatient hospitalization or prolongation of existing hospitalization, results in persistent or significant disability or incapacity, or causes a congenital anomaly or birth defect.

The study design includes an independent efficacy and safety assessment committee that will review the ongoing safety data in an unblinded manner in accordance with the Standard Operating Procedures for Clinical Trials, Japan Medical Association (http://www.jmacct.med.or.jp/).

### 2.8 Outcome

The primary endpoint of this study is the achievement rate of >1 improvement in CHAP score from baseline at 16 weeks of study drug administration. The secondary endpoints are efficacy, safety, and exploratory categories.

#### 2.8.1 Efficacy

We will evaluate the efficacy of the investigational drug based on the following parameters:

1) Hb (g/dL): change from baseline at 2, 4, 8, 12, and 16 weeks, and at the time of drug discontinuation.
2) Alb (g/dL): change from baseline at 2, 4, 8, 12, and 16 weeks, and at the time of drug discontinuation.
3) CRP (mg/dL): change from baseline at 2, 4, 8, 12, and 16 weeks, and at the time of drug discontinuation.
4) Physician global assessment (disease activity assessment, 100 mm visual analog scale [VAS]): change from baseline at 2, 4, 8, 12, and 16 weeks, and at the time of drug discontinuation.
5) Patient global assessment (disease activity assessment, 100 mm VAS): change from baseline at 2, 4, 8, 12, and 16 weeks, and at the time of drug discontinuation.
6) SF-36: change from baseline at 4, 8, 12, and 16 weeks, and at the time of drug discontinuation.
7) Lymph node changes in subjects with lymphadenopathy: change from baseline at 16 weeks after initiation of the study drug initiation, change from baseline at the time of the drug discontinuation, as well as change in the number of lymph nodes ≥15 mm in diameter from baseline at 16 weeks after initiation of the study drug, at time of discontinuation.
8) CHAP score: change from baseline at 2, 4, 8, 12, and 16 weeks, and at the time of drug discontinuation.
9) CHAP score minus CRP score: change from baseline at 2, 4, 8, 12, and 16 weeks, and at the time of drug discontinuation.
10) CR achievement, defined by the Castleman Disease Collaborative Network (CDCN), response criteria^[9]^ at 16 weeks and at the time of drug discontinuation.
11) PR achievement, defined by the CDCN response criteria^[9]^ at 16 weeks and at the time of drug discontinuation.

#### 2.8.2 Safety

The safety evaluation indices of this clinical trial are as follows:

1) Adverse events, including adverse event incidence rate, serious adverse event incidence rate, and side effect incidence rate.
2) Laboratory tests, including hematological examination, blood biochemical examination, and urinalysis.
3) All medically important indicators, including physical findings, vital signs, imaging tests, electrocardiogram results, echocardiographic findings, etc.
4) Pharmacodynamic assessment, including the measurement of the serum sirolimus level.

#### 2.8.3 Exploratory

Further research will be conducted to analyze the relationship between the efficacy, safety, and pharmacokinetics of the investigational drug and serum proteins, as well as the relationship between the progression of the disease and prognosis and changes in serum proteins before and after treatment. In preparation for the conduct of the study, the remaining samples (at the time of screening; at 2, 4, 8, 12, and 16 weeks; and at the time of discontinuation of the drug) will be tested for serum protein arrays with the subject's consent, separately from the trial's consent. In addition, we will collect blood samples for a pharmacogenomics study under the consent of the patient separately from the consent of this trial.

### 2.9 Data collection and management

The investigators will be provided access to an online, web-based, EDC system. Only the investigator will be able to enter and modify data in the electronic CRF. All study findings and documents will be regarded as confidential. Patients will be identified on the electronic CRF by their patient number, but not by name. Confidentiality of the documents that identify the patient must be maintained by the investigator to ensure the anonymity of the participants. The study blind should only be broken in a medical emergency, such as cases in which knowledge of the study drug received would affect the emergency treatment, or as a regulatory requirement (e.g., for serious adverse events or death).

During the study, a sponsor-investigator will perform regular site visits to review protocol compliance, conduct source data verification, assess drug accountability and management, assess laboratory procedures, and ensure that the study is being conducted according to pertinent regulatory and protocol requirements. The sponsor-investigator controls the quality of the trial in accordance with the protocol, each standard operating procedure, and the trial monitoring/audit protocol. The primary operations are as follows:

1) Hold and explain the protocol to the investigator or trial collaborators at the start of the trial or as necessary to ensure an accurate understanding of the protocol and standardization of judgment and evaluation.
2) Eligibility of subjects will be automatically checked by EDC system to eliminate selection and exclusion violations.
3) The monitor should visit the institution on a regular basis to ensure that the protocol and good clinical practices are followed for the proper conduct of the trial and to confirm the accuracy of the data.
4) Records and reports concerning the operation of clinical trials, data collection, data management, statistical analysis, and analysis of adverse events shall be performed in accordance with standard operating procedures, and inspections and confirmations shall be made.
5) In order to ensure that the clinical trial is conducted properly, the sponsor-investigator will ensure the quality of each development activity through quality control. Auditors independent from departments involved in the development and implementation of drugs will systematically investigate and confirm clinical trial operations and documents in accordance with standard operating procedures for the implementation of audits.
6) All source documents will be made available for review at the request of the sponsor-investigator or national or international regulatory authority.

### 2.10 Sample size considerations

According to a survey by the Research Group for Intractable Diseases funded by the National Institute of Health, Labour and Welfare in Japan, it has been reported that the number of patients with iMCD in Japan is approximately 1500. Among 149 patients with iMCD investigated in the research group, TCZ was administered to 81 patients. The effect of TCZ was determined to be insufficient in 37 patients (45.6%). Assuming that the percentage of patients who can obtain consent is 80%, the maximum number of patients in the research group will be 30; considering the limited number of research institutions, we set the number of cases for this study at 20.

### 2.11 Statistical analysis

The full analysis set (FAS) will consist of all randomized and treated patients for whom 1 or more efficacy endpoints could be evaluated. The per-protocol set will consist of patients in the FAS excluding those with major protocol violations. We will perform statistical analyses for the primary endpoint using the FAS and for the secondary endpoints using both the FAS and per-protocol set.

The effect of the allocation group on the primary endpoint will be analyzed by a logistic regression model using the baseline CHAP score as a covariate, and the following null hypothesis will be tested at a significance level of 0.05 (Wald test, regression coefficient of the allocation group = 0). For the analysis of secondary endpoints, the effects on the time course of the treatment group will be analyzed as a regression coefficient of the interaction terms between the treatment group and the time point, using a mixed effects model in which the baseline CHAP score will be included as fixed effects and individual subjects’ IDs as a random effect.

The safety analysis set will consist of all patients who receive at least 1 dose of the study drug. The safety and tolerability analyses will be based on the safety analysis set. We will replace adverse events with the corresponding MedDRA code and tabulate the number of expression cases and the number of expression cases for each event defined by the system organ class and preferred term. We will summarize the number of adverse events by system organ class and preferred term. In addition, the number of cases should be tabulated separately by causality, severity, and seriousness. For continuous endpoints, a case transition diagram should be prepared for each endpoint. We will calculate summary statistics for each endpoint at each time point.

## 3 Discussion

The objective of this study is to evaluate the safety and efficacy of sirolimus (2 mg/d) in patients with TCZ-resistant iMCD. In order to properly evaluate the efficacy of sirolimus, placebo will be used as a double-blind, parallel-group comparative study. The basis for setting the amount of change in CHAP score as the primary endpoint in this study follows. The efficacy of TCZ in iMCD has been evaluated in an open-label trial in Japan using inflammatory markers (CRP, fibrinogen, and erythrocyte sedimentation rate), with general malaise measured using VAS, Hb, and hypoalbuminemia.^[20]^ The CHAP score was proposed as an index to evaluate the disease activity of iMCD,^[10]^ The components of the CHAP score are CRP, Hb, Alb, and Eastern Cooperative Oncology Group Performance Status, which are similar to the evaluation items used to evaluate the effectiveness of TCZ. It is considered sufficiently possible to evaluate the effectiveness of sirolimus using this method. The CDCN has established response criteria consisting of 3 components: biochemical markers of inflammatory response and organ function, lymph node size, and clinical symptoms to assess response taking into account all key features of iMCD.^[9]^ We determined that an assessment based on these international criteria was also necessary and, thus, set the CDCN response criteria achievement rate as a secondary endpoint.

Sirolimus was approved for treatment lymphangioleiomyomatosis (LAM) in Japan in 2014, and the long-term safety profile of sirolimus has been shown previously.^[21]^ We set the dosage of the investigational drug at 2 mg orally once daily in accordance with the approved dosage for LAM in Japan. An international study (MILES trial)^[22]^ and a Japanese clinical trial (MLSTS trial)^[21]^ in patients with LAM have shown that most of the patients treated with 2 mg of this drug maintained a trough blood concentration in the range of 5 to 15 ng/mL. These findings suggest that this dose suppresses the constitutive activity of mTOR, and that it is an acceptable and effective dose for patients with iMCD. Accordingly, dose adjustment using trough level was not required in this trial.

This trial will evaluate the efficacy and safety of sirolimus in patients with TCZ-resistant iMCD. It will support the potential efficacy of mTOR inhibitor on preventing organ damage and secondary amyloidosis in iMCD with inadequate TCZ response and on significantly improving quality of life and vital prognosis. The findings of this study are expected to provide a new treatment option for severe iMCD.

### 3.1 Trial status

The trial started on January 1, 2020, and is currently recruiting participants.

## Acknowledgments

The authors would like to thank their colleagues and staff at the Rheumatology Department of Nagasaki University Hospital for their support.

## Author contributions

TK, NH, HY and AK are responsible for conceiving and designing the trial, planning data analysis, drafting the manuscript, making the final decision to terminate the trial, and approving the final manuscript. TK, NH, ST, RS, TS, and CF will participate in data collection and are in charge of recruitment and treatment of patients. TK, NH, and SM are responsible for planning data analysis and analyzing the data resulting from the trial. All authors will have access to the interim results as well as the capacity to discuss, revise, and approve the final manuscript.
